# Accurate and stable two-step LED position calibration method for Fourier ptychographic microscopy

**DOI:** 10.1117/1.JBO.26.10.106502

**Published:** 2021-10-15

**Authors:** Haojie Wei, Jing Du, Lei Liu, Yu He, Yong Yang, Song Hu, Yan Tang

**Affiliations:** a Institute of Optics and Electronics Chinese Academy of Sciences, State Key Laboratory of Optical Technologies for Nano-Fabrication and Micro-Engineering, Chengdu, China; b University of Chinese Academy of Sciences, Beijing, China

**Keywords:** Fourier ptychographic microscopy, computational imaging, positional misalignment correction

## Abstract

**Significance:** Fourier ptychography microscopy (FPM) is a computational optical imaging technology that employs angularly varying illuminations and a phase retrieval algorithm to achieve a wide field of view and high-resolution imaging simultaneously. In the FPM, LED position error will reduce the quality of the reconstructed high-resolution image. To correct the LED positions, current methods consider each of the LED positions as independent and use an optimization algorithm to get each of the positions. When the positional misalignment is large or the search position falls into a local optimal value, the current methods may lack stability and accuracy.

**Aim:** We improve the model of the LED position and propose an accurate and stable two-step correction scheme (tcFPM) to calibrate the LED position error.

**Approach:** The improved LED positions model combines the overall offset, which represents the relative deviation of the LED array and the optical axis, with the slight deviation of each LED’s independent position. In the tcFPM, the overall offset of the LED array is corrected at first, which obtains an approximate value of the overall offset of the LED array. Then the position of each LED is precisely adjusted, which obtains the slight offset of each LED.

**Results:** This LED position error model is more in line with the actual situation. The simulation and experimental results show that the method has high accuracy in correcting the LED position. Furthermore, the reconstruction process of tcFPM is more stable and significantly improves the quality of the reconstruction results, which is compared with some LED position error correction methods.

**Conclusions:** An LED position error correction technology is proposed, which has a stable iterative process and improves the reconstruction accuracy of complex amplitude.

## 1 Introduction

As is well known, there is a contradiction between the high resolution (HR) and wide field of view (FOV) in conventional microscopy, due to limited physical space-bandwidth-product (SBP). Therefore, it is difficult to get HR with wide FOV in the conventional microscope. To solve this problem, a computational super-resolution imaging method known as Fourier ptychography microscopy (FPM) has been proposed.^1^^–^^3^ In FPM, a programmable LED array is used to provide illumination with varying angles. When the LEDs are turned on sequentially, the low-resolution (LR) images of the sample with the corresponding illumination angle are collected from a wide FOV objective lens.^4^^,^^5^ After that, the corresponding spectra of the LR image sequence are stitched through an iterative phase recovery process.^6^ In this way, FPM can obtain the HR and wide FOV complex amplitude of the sample.

In FPM, there are many system error sources such as aberrations,^7^^,^^8^ noise,^9^^–^^12^ vignetting effect,^13^ periodic gratings caused by LED arrays,^14^ LED intensity fluctuation, ^15^^,^^16^ and LED misalignment,^17^^–^^25^ resulting in serious degradation of the reconstruction quality of the FPM. Within these systematic errors, the LED position error is of great importance to the reconstruction quality. Because in the process of iterative high-resolution spectral splicing, the position coordinates of the LED are used to calculate the wave vector of the illumination, the misalignment of the LED position will bring serious errors to the reconstruction result.

LED position error correction is very important to improve the quality of reconstructed HR images. Different position calibration schemes have been proposed for the problem of LED misalignment. In most of those methods, the position of each LED is considered independent, and optimization algorithm is used to get each LED position. The simulated annealing (SA) is used to correct the position error of each LED in the spatial domain.^17^ The SA is a local optimization algorithm, which causes confusion in the reconstructed LED position coordinates. The position of each LED is also corrected in the frequency domain.^18^ This method uses Newton’s method (rather than gradient descent method) to correct the position error, which enhances the convergence rate. If the positional misalignment is large or the search position falls into the local optimal value, the algorithm may not be able to get satisfactory results. The rpcFPM was proposed to correct the random positional deviations of each LED element with a feedback parameter and objective function constraint based on ePIE algorithm.^19^ However, this scheme ignores the influence of the overall error of the LED matrix on the reconstruction quality. Moreover, since the performance is limited by the ePIE algorithm, rpcFPM may only reach a local optimum if each LED has large deviations.

To improve the correction stable and accuracy of LED position errors, many methods have been presented. A correction scheme called BFL was proposed,^20^ which estimates the global position error parameters by accurately locating the bright field area on the sample plane. This correction scheme can correct the global offset of the LED array, but the personal positional error of the LED is ignored. Another self-calibration algorithm that uses circular edge detection to calculate the position error of the bright field LED was proposed.^21^ It is limited by the spectral distribution of the sample and the imaging noise of the system. The mcFPM method divides the LED array into many segments and correct the global offset of each small segment.^22^ This method introduces the magnification in the error model to correct the aberration of the imaging system. By adjusting the mechanical structure of the FPM system, the impact of the misalignment of the LED array and the optical axis is reduced. But the relative offset between LEDs is not considered in this method. A calibration scheme based on SA algorithm and nonlinear regression technology was proposed by Sun et al.,^23^ which is called pcFPM.^24^ They introduced a global position misalignment model for LED array position correction, which solves the problem of LED position disorder after SA algorithm correction. The pcFPM method first uses the SA algorithm to correct the position of each LED in the central area, and then it obtains the initial solution of the global position model through nonlinear regression. However, after the global error of LED position is corrected by nonlinear programming, the reconstruction result may be degraded, and the small offset between LEDs is also ignored. Generally, in the process of designing the LED array, the position of the PCB pad is fixed, but the LED will have a slight position shift during the soldering process. This position offset is smaller than the solder pad pattern size. In pcFPM, the global calibration model will amplify the influence of the minute offset between LEDs, which will obviously have an impact on the improvement of reconstruction quality.

In this paper, we propose a correction scheme, termed two-step correction FPM (tcFPM). Inspired by the different sources of LED position error, we improve the LED model by combining the overall offset, which represents the relative deviation of the LED array and the optical axis, with the slight deviation of each LED’s independent position. We correct the LED position error into two types for stepwise correction: the first is the position error between LED array design and production, which is called the slight offset between LEDs, and the second is the position error between the LED array and the optical axis of the imaging system, which is called the global offset of the LED array. Compared with existing schemes, this LED position error model is more in line with the actual situation. First, the global offset of the LED array is calculated during the initial calibration. Then, the slight shift between LEDs is obtained through the precise calibration process. Compared with pcFPM, the correction scheme proposed by tcFPM can ensure the accuracy of LED array position. It avoids the influence of the global optimization model on the reconstruction quality and improves the reconstruction accuracy. The correction of the slight offset between LEDs is performed on the basis of the global correction, which further improves the imaging accuracy of the system. Furthermore, the reconstruction process of tcFPM is more stable and significantly improves the quality of the reconstruction results. Both simulations and experiments show that the tcFPM has better accuracy and stability for LED array position error correction.

The following parts of this article are arranged as follows. In Sec. 2, the principle of the proposed method is introduced, including the analysis of FPM imaging theory and LED position error types in Secs. 2.1 and 2.2. Section 2.3 introduces the tcFPM algorithm flow in detail. In Secs. 3.1 and 3.2, simulations and experiments are respectively elaborated to prove the effectiveness of the tcFPM. Finally, in Sec. 4, the work of this article is summarized.

## 2 Method

### 2.1 Forward Imaging of the FPM

**Fig. 1:**
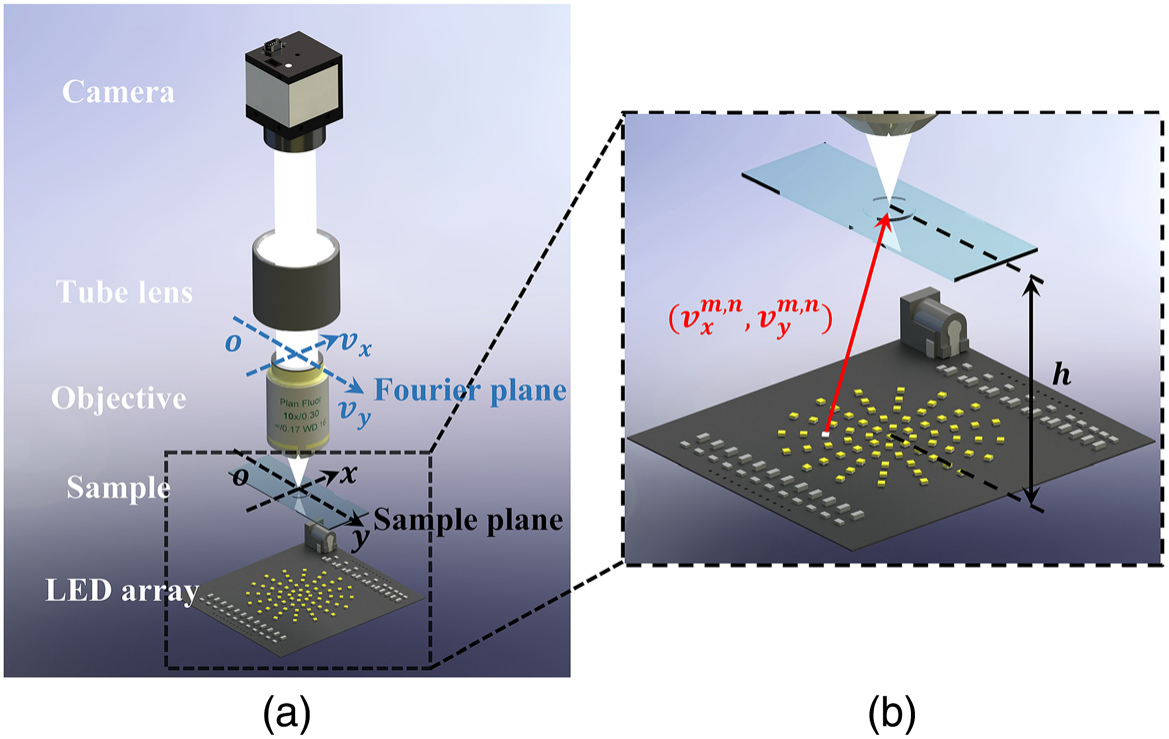
Schematic diagram of a typical FPM imaging process. (a) The classic setup of FPM. (b) The illumination wave-vector of the LED located in the circle.

To demonstrate the importance of LED position error correction, the basic concepts of FPM imaging model are introduced. The typical FPM system setup is shown in Fig. 1(a), which mainly includes CCD, tube lens and low NA microscope objective lens, sample, and the LED array. The LED array is shown in Fig. 1(b), which is an enlarged view of the parts within the black dotted box in Fig. 1(a). The distance between LEDs and sample is h. If the light emitted by the LED is an ideal plane wave, and its incident wave vector $v_{x}^{m,n},v_{y}^{m,n}$ can be expressed as

$$v_{x}^{m,n}=-\frac{2\pi }{\lambda }\frac{x_{0}-x_{m,n}}{\sqrt{(x_{0}-x_{m,n}{)}^{2}+(y_{0}-y_{m,n}{)}^{2}+h^{2}}}\;v_{y}^{m,n}=-\frac{2\pi }{\lambda }\frac{y_{0}-y_{m,n}}{\sqrt{(x_{0}-x_{m,n}{)}^{2}+(y_{0}-y_{m,n}{)}^{2}+h^{2}}}$$ (1)

The LEDs are turned on sequentially to realize the illumination of the sample from various incident angles. Synchronously, the LR intensity image of the sample illuminated at the corresponding angle is collected by the CCD. This is explained further using mathematics. In this paper, it is assumed that the sample is very thin. When the LED located at (m,n) the is turned on, under the action of its illumination wave vector$v_{x}^{m,n},v_{y}^{m,n}$, the complex amplitude of the sample iso(x,y)written as

$$S_{\text{output}}(x,y)=o(x,y)\cdot e^{iv_{x}^{m,n}x+iv_{y}^{m,n}y},$$ (2)

where $S_{\text{output}}(x,y)$ represents the output complex amplitude of the sample, and (x,y)is the coordinate system in the sample plane. Then, the complex amplitude passes through the microscope objective, and this process in the Fourier plane is given as

$$F_{\text{output}}(v_{x},v_{y})=F\{S_{\text{output}}(x,y)\}\cdot P(v_{x},v_{y})=O(v_{x}-v_{x}^{m,n},v_{y}-v_{y}^{m,n})\cdot P(v_{x},v_{y}),$$ (3)

where $F_{\text{output}}(v_{x},v_{y})$ denotes the sample spectrum, F is the Fourier transform operator, the coherent transform function of the imaging system is represented by $P(v_{x},v_{y})$, and the $O(v_{x}-v_{x}^{m,n},v_{y}-v_{y}^{m,n})$ stands for the sample spectrum. Accordingly, the CCD captures the LR intensity image of the sample, the formula is as follows:

$$I_{\mathrm{captured}}^{m,n}(x,y)=|F^{-1}\{O(v_{x}-v_{x}^{m,n},v_{y}-v_{y}^{m,n})\cdot P(v_{x},v_{y})\}{|}^{2}$$ (4)

where $F^{-1}$ represents the inverse Fourier transform.

When the LED in different positions is turned on, the LR intensity image sequence of the corresponding samples is saved. In this way, the sample spectra in different sub-regions can bypass the physical limitations of the imaging system. Then, the FPM reconstruction algorithm that iteratively switches between the spatial domain and the Fourier domain stitches these spectra together correctly. After iterative convergence, the high-resolution complex amplitude result of the object is obtained.

### 2.2 Analysis of LED Positional Misalignment

In the FPM iterative reconstruction process, it is necessary to match the space position coordinates of the real LED and the sub-aperture spectrum position coordinates. Therefore, the position coordinates of the LED directly affect the accuracy of spectrum splicing and determine the quality of the complex amplitude of the reconstructed sample. There are two sources of LED position error: the first one is the deviation between the actual LED position and the design position produced during the manufacturing process, and the second is the relative position error of the LED array and the optical axis during installation. The offset between LEDs is a tiny random variable that cannot be ignored, which will increase the uncertainty of the reconstruction result. The latter is the global offset of the LED array, which will cause the quality of the reconstruction result to drop rapidly.

**Fig. 2:**
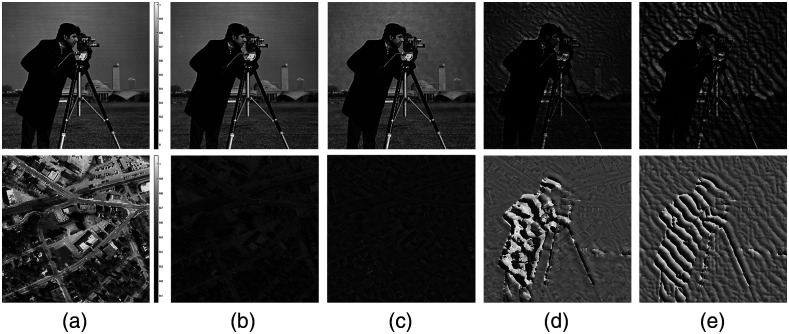
The simulation results of LED position misalignment in the FPM. (a) The original HR amplitude and phase images, respectively. (b) Without LED position error, the HR amplitude and phase graphics restored by traditional algorithms. (c) The reconstructed amplitude and phase image with the slight offset between LEDs. (d) In the case of the relative position error of the LED lighting module and the optical axis, the reconstructed intensity and phase image. (e) The reconstructed intensity and phase simulation results, include (c) and (d) the two LED position errors.

Figure 2 shows the simulated amplitude and phase results of LED position error, respectively. The high-resolution amplitude and phase images used in the simulation are shown in Fig. 2(a). Figure 2(b) shows the reconstructed image without LED position error, which is not much different from Fig. 2(a). As mentioned above, there are two types of LED position error. Figure 2(c) is the reconstructed amplitude and phase image with the slight offset between LEDs. It can be seen that this tiny, random LED position variable will have a corresponding impact on the reconstruction quality. In addition, the reconstructed amplitude and phase image, which is demonstrated in Fig. 2(d), corresponds to the second type of LED position error. The relative position error of the LED array and the optical axis will cause the global shift of the spectrum, which will have a great influence on the reconstruction result. The combined effect of these two errors is shown in Fig. 2(e), which can lead to worse reconstruction results of intensity and phase. Thus, it is necessary to correct these two types of position errors in FPM.

### 2.3 Proposed Misalignment Correction Algorithm

**Fig. 3:**
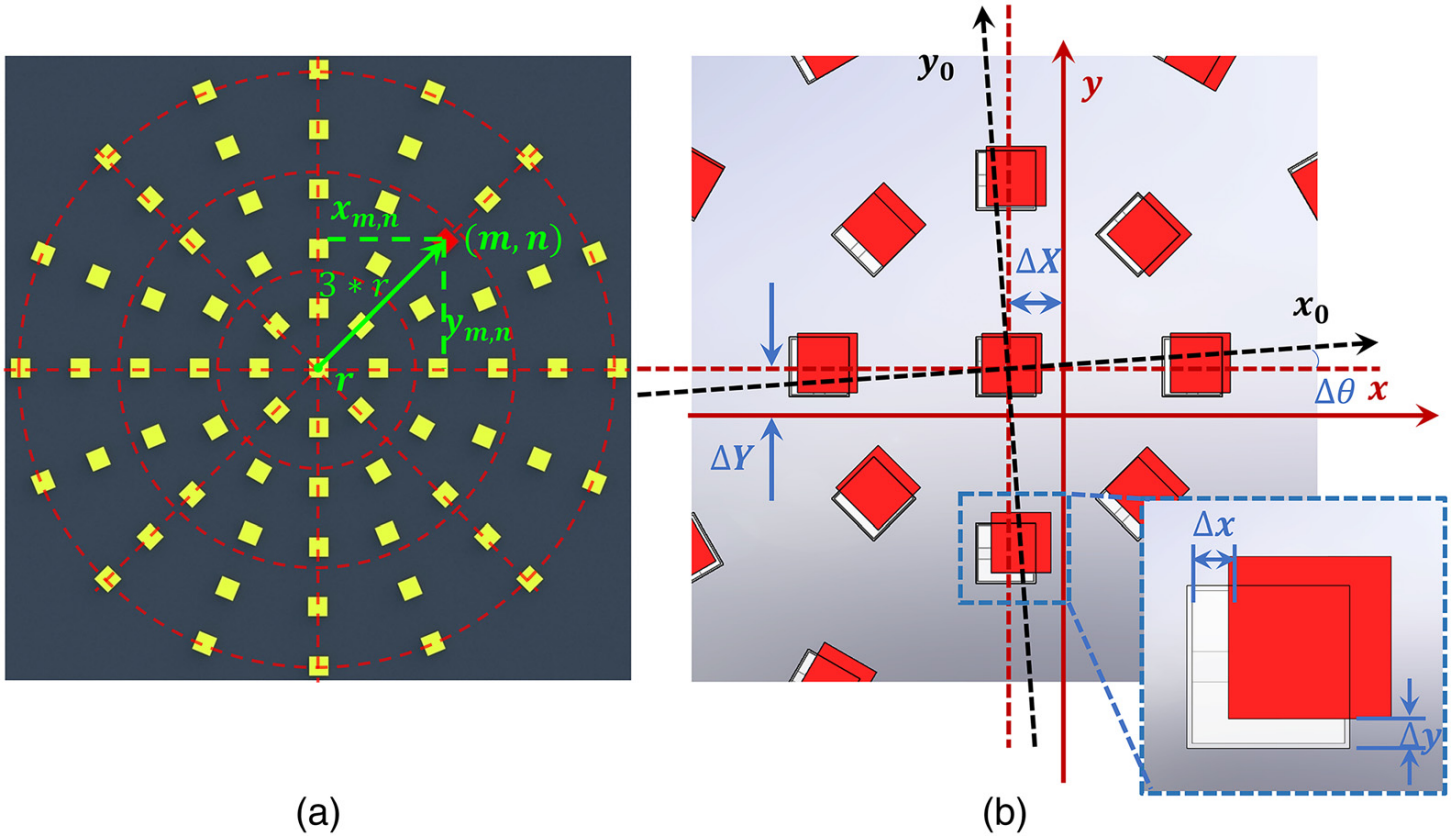
The position distribution model of the LED array. (a) The schematic diagram of the nonlinear distribution of LEDs. (b) The parameter representation of the LED array misalignment model.

Before introducing the tcFPM algorithm process, the position distribution model of the LED array is defined. In the simulation and experiment of this paper, the LED array is a non-linear annular distribution, which can effectively avoid the pathological problem of the grid,^26^^,^^27^ as shown in Fig. 3(a). The LED array is composed of a central LED and five LED rings. The number of LEDs included in each LED ring is N, where N∈{8,12,16,16,16}. The distance between two adjacent LED rings is r=5  mm. For example, the LED marked in red in Fig. 3(a), which is located at the m=3(m∈{1,2,3,4,5}), n=3(n∈{1,2,3,4,5,6,7,8,9,10,11,12}). In Fig. 3(b), the black dashed axis represents the ideal space coordinates, and the ideal LED spatial distribution is demonstrated by white transparent LEDs. The real coordinates and the spatial distribution of LEDs are indicated by the red solid line coordinate axis and the red transparent LED. The $\Delta x_{m,n}$ and $\Delta y_{m,n}$, respectively, represent the deviation of the LED position in two directions. In addition, the displacement error of the relative position of the LED array and the optical axis is distinguished by Δθ,ΔX, and ΔY. Therefore, the coordinates of the LED including the deviation of the LED position and the error of the relative position of the LED array and the optical axis can be expressed as

$$x_{m,n}=m*r*\sin(\frac{2\pi }{N}*n+\Delta \theta )+\Delta X+\Delta x_{m,n}\;y_{m,n}=m*r*\cos(\frac{2\pi }{N}*n+\Delta \theta )+\Delta Y+\Delta y_{m,n}.$$ (5)

**Fig. 4:**
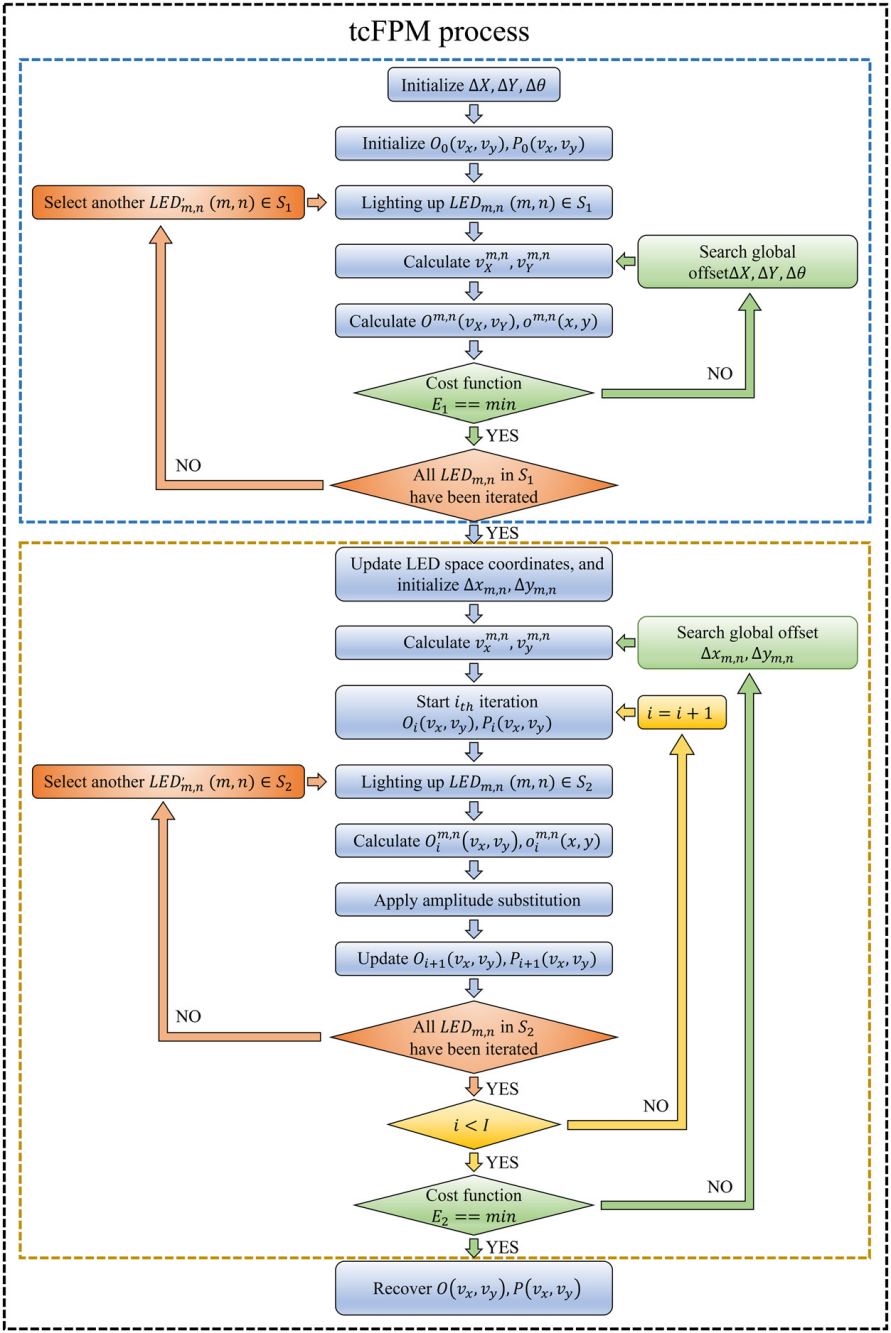
The flow chart of tcFPM method.

Aiming at the above two different LED position errors, we propose tcFPM, which is a system scheme for initial correction to obtain the global offset of the LED array, and then precise correction to obtain the slight offset between LEDs. The algorithm flow chart of tcFPM is illustrated in Fig. 4.

The algorithm process is mainly composed of two parts: the blue dashed frame in Fig. 4 is the correction process of the global offset of the LED array, and the correction process of the slight offset between LEDs is marked with the brown dashed frame. The LR image corresponding to the LED array in the bright field range contains a large amount of sample information, which has a greater impact on the image quality of FPM. Therefore, First, the global offset of the LED array in the bright field is preliminarily corrected. In this article, the initial correction LED range, which includes a center LED and two adjacent LED rings. The reason why the range is selected is to be able to achieve a large-scale global correction capability and to ensure the robustness of the algorithm.

First, the global error variable Δθ,ΔX,ΔY, the Fourier spectrum of the HR sample image $O_{0}(v_{x},v_{y})$and the pupil function $P_{0}(v_{x},v_{y})$are initialized. Generally, the initial value of the global error variable is set toΔθ=0,ΔX=0,ΔY=0. When the bright field LEDs are simultaneously illuminated, the corresponding sample image is collected. The obtained sample image is subjected to bilinear interpolation operation, and then the result after Fourier transform is used as the initial sample spectrum $O_{0}(v_{x},v_{y})$. The pupil function $P_{0}(v_{x},v_{y})$ is initialized as a circular low-pass filter, and the radius of the circle is2πNA/λ, where NA is the numerical aperture of the brightfield microscope objective and λ is the central wavelength of the illumination.

Next, the incident wave vector $v_{X}^{m,n},v_{Y}^{m,n}$ is calculated according to Eqs. (1) and (5), which includes the LED global offset errorΔθ,ΔX,ΔY. Based on Eqs. (2) and (3), the spectrum information$\Psi^{m,n}(v_{x},v_{y})$and intensity information $\psi^{m,n}(x,y)$ of the sample after the objective pupil function can be obtained as follows:

$$\Psi^{m,n}(v_{x},v_{y})=O^{m,n}(v_{x}-v_{X}^{m,n},v_{y}-v_{Y}^{m,n})\cdot P(v_{x},v_{y})\;\psi^{m,n}(x,y)=F^{-1}\{O^{m,n}(v_{x}-v_{X}^{m,n},v_{y}-v_{Y}^{m,n})\cdot P(v_{x},v_{y})\}.$$ (6)

Further, we compare the calculated intensity distribution $o^{m,n}(x,y)$ with $I_{S_{1}}^{m,n}(x,y)$ using the following formula to find the smallest cost function $E_{1}$.

$$E_{1}=\min_{\Delta X,\Delta Y}\sum_{m,n}\sum_{x,y}(I_{S_{1}}^{m,n}(x,y)-|\psi^{m,n}(x,y){|}^{2}{)}^{2}$$ (7)

where, $I_{S_{1}}^{m,n}(x,y)$represents the LR image corresponding to the LED at position (m,n) within the range of $S_{1}$. Since Δθ,ΔX,ΔY is close to the relative offset error of the LED and the optical axis under real conditions, the value of $E_{1}$ should be the smallest. To not only minimize the cost function $E_{1}$, but also compare with the existing methods, the SA algorithm is used to find Δθ,ΔX,ΔY.

The brown dashed box in Fig. 4 shows the process of correcting the slight offset between LEDs. The spatial position coordinates of the LED are updated, which is uses the global offset obtained by the preliminary calibration. The incident wave vector $v_{x}^{m,n},v_{y}^{m,n}$ including the LED random position deviation $\Delta x_{m,n},\Delta y_{m,n}$ is calculated according to expressions Eqs. (1) and (5). Before the iteration starts, the Fourier spectrum $O_{i}(v_{x},v_{y})$ of the high-resolution sample and the pupil function $P_{i}(v_{x},v_{y})$ need to be initialized. Similarly, the spectrum information $\Psi^{m,n}(v_{x},v_{y})$ and intensity information $\psi^{m,n}(x,y)$ of the sample after the objective pupil function can be written as:

$$\Psi^{m,n}(v_{x},v_{y})=O_{i}^{m,n}(v_{x}-v_{x}^{m,n},v_{y}-v_{y}^{m,n})\cdot P_{i}(v_{x},v_{y})\;\psi^{m,n}(x,y)=F^{-1}\{O_{i}^{m,n}(v_{x}-v_{x}^{m,n},v_{y}-v_{y}^{m,n})\cdot P_{i}(v_{x},v_{y})\}.$$ (8)

Further, following the principle of phase retrieval, the phase information of the target image remains unchanged. The amplitude component of the target image is replaced by the square root of the actual measurement value. In this way, the updated LR target image $\phi_{i}^{m,n}(x,y)$ and its Fourier spectrum $\Phi_{i}^{m,n}(x,y)$ are calculated as follows:

$$\phi_{i}^{m,n}(x,y)=\sqrt{I_{S_{2}}^{m,n}(x,y)}\frac{\psi^{m,n}(x,y)}{|\psi^{m,n}(x,y){|}^{2}}\;\Phi_{i}^{m,n}(v_{x},v_{y})=F^{-1}\{\sqrt{I_{S_{2}}^{m,n}(x,y)}\frac{\psi^{m,n}(x,y)}{|\psi^{m,n}(x,y){|}^{2}}\}$$ (9)

where the corresponding LR image of the LED located at the (m,n) in the range of $S_{2}$ is represented by $I_{S_{2}}^{m,n}(x,y)$.

Next, we update the spectral information in the corresponding sub-aperture in the HR spectrum of the object and the pupil function:^25^

$$O_{i+1}(v_{x},v_{y})=O_{i}(v_{x},v_{y})+\alpha \frac{\left|P_{i}(v_{x}+v_{x}^{m,n},v_{y}+v_{y}^{m,n})|\cdot P_{i}^{*}(v_{x}+v_{x}^{m,n},v_{y}+v_{y}^{m,n}\right)}{\left|P_{i}(v_{x},v_{y}){|}_{\max}(|P_{i}(v_{x}+v_{x}^{m,n},v_{y}+v_{y}^{m,n}){|}^{2}+\delta_{1}\right)}\;\cdot \{\Phi_{i}(v_{x}+v_{x}^{m,n},v_{y}+v_{y}^{m,n})-O_{i}(v_{x},v_{y})\cdot P_{i}(v_{x}+v_{x}^{m,n},v_{y}+v_{y}^{m,n})\}\;P_{i+1}(v_{x},v_{y})=P_{i}(v_{x},v_{y})+\beta \frac{\left|O_{i}(v_{x}-v_{x}^{m,n},v_{y}-v_{y}^{m,n})|\cdot O_{i}^{*}(v_{x}-v_{x}^{m,n},v_{y}-v_{y}^{m,n}\right)}{\left|O_{i}(v_{x},v_{y}){|}_{\max}(|O_{i}(v_{x}-v_{x}^{m,n},v_{y}-v_{y}^{m,n}){|}^{2}+\delta_{2}\right)}\;\cdot \{\Phi_{i}(v_{x},v_{y})-O_{i}(v_{x}-v_{x}^{m,n},v_{y}-v_{y}^{m,n})\cdot P_{i}(v_{x},v_{y})\},$$ (10)

where, $\delta_{1}$ and $\delta_{2}$ are regularization constants, which are used to ensure numerical stability. Set them as $\delta_{1}=1$, $\delta_{2}=1000$in the tcFPM. In addition, α and β are the coefficient parameters related to the step length in the iteration process, and they are both set to 1 in this work. Repeat the above process until the reconstruction algorithm converges.

Finally, the calculated intensity distribution $\psi^{m,n}(x,y)$ is compared with $I_{S_{2}}^{m,n}(x,y)$ using the following formula. The cost function for finding LED position error $\Delta x_{m,n},\Delta y_{m,n}$ is expressed as

$$E_{2}=\min_{\Delta x,\Delta y}\sum_{m,n}\sum_{x,y}(I_{S_{2}}^{m,n}(x,y)-|\psi^{m,n}(x,y){|}^{2}{)}^{2}$$ (11)

where, $I_{S_{2}}^{m,n}(x,y)$ indicates the LR image corresponding to the LED located at (m,n) in the range of $S_{2}$. Similarly, the SA algorithm is used to search for the $\Delta x_{m,n},\Delta y_{m,n}$ in tcFPM. The purpose is to minimize the cost function $E_{2}$ while comparing with existing methods. When correcting the slight offset between LEDs, the search direction is set to $\Delta x_{m,n},\Delta y_{m,n}$. The size of the LED is1.6  mm×1.6  mm, and the solder pad pattern size recommended by the manufacturer is1.7  mm×1.7  mm. Therefore, the search range of the LED offset in this system is set to [−2  mm,2  mm]. This can avoid the confusion of the LED position during the calibration process, and another advantage is that it simplifies the search process.

The tcFPM algorithm carries out a two-step correction process according to the difference of LED position error, which has good stability. The problem of the LED position error is solved, and a better high-resolution reconstruction result of the sample can be obtained. The simulation in Sec. 3.1 and the experiment in Sec. 3.2 will prove that the reconstruction results of tcFPM can be more accurate and stable for a set of LR images containing position errors.

## 3 Results

### 3.1 Simulation

In this section, the effectiveness of the tcFPM scheme is further illustrated by calculation simulation. The system parameters used in the simulation are consistent with the component parameters adopted in the experiment. The NA of the microscope objective is 0.3, and its magnification is 10. The pixel size of the CCD is2.75  μm, and the simulated image size is 512×512  pixels. The center wavelength of the LED is set to 632 nm, and its spatial distribution is consistent with the model mentioned above. The distance between the LED array plane and the sample is 80 mm. The slight offset between LEDs and the global offset of the LED array are simulated separately. These two errors are represented by random variables uniformly distributed within a certain range, to ensure the adaptability and stability of the tcFPM algorithm. The reconstruction process of simulation and experiment are performed in MATLAB R2020a.

**Fig. 5:**
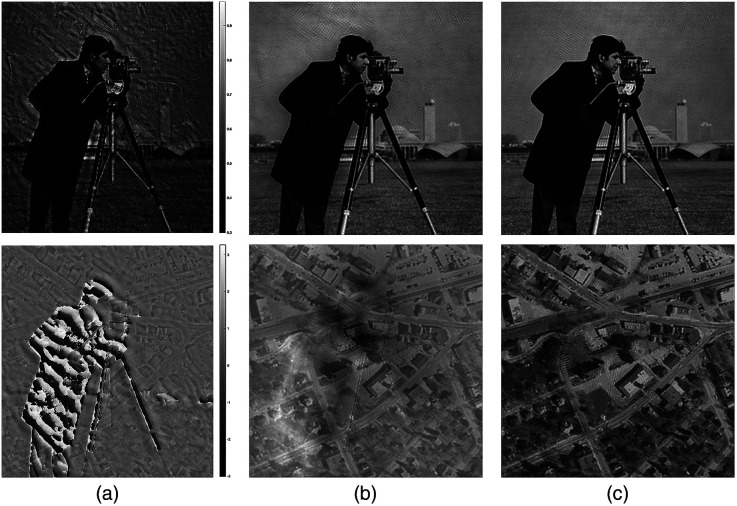
The Results of simulation recovery of different algorithms. (a) The intensity and phase images restored by traditional FPM. (b) The intensity and phase images reconstructed with pc-FPM. (c) Intensity and phase images calculated with tcFPM.

The intensity and phase images of the sample used in the simulation are shown in Fig. 2(a). In the simulation, 69 LR intensity images are used. During the reconstruction of tcFPM, the number of iterations of the global correction process is 6, and the number of iterations of the precise correction process is 14. The reconstruct results of the traditional FPM, pcFPM scheme and tcFPM are demonstrated in Figs. 5(a), 5(b), and 5(c), respectively. It is shown that with the same LED array position error, the high-resolution intensity image and phase recovered by the tcFPM proposed in this paper have more details.

**Fig. 6:**
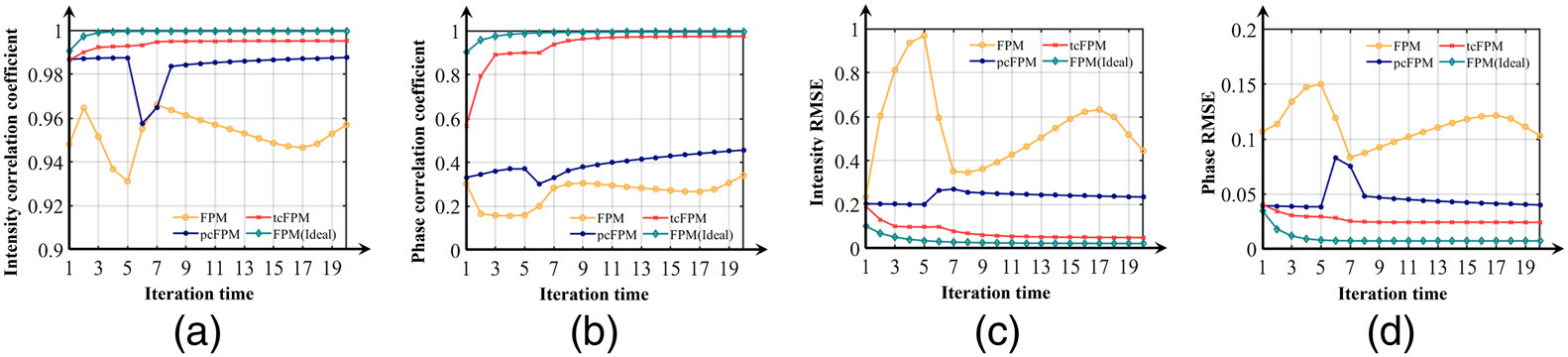
(a) The intensity correlation coefficient between the restored complex amplitude image and the original high-resolution image, corresponding to four different reconstruction iteration processes. (b) The phase correlation coefficient between the restored complex amplitude image and the original high-resolution image, corresponding to four different reconstruction iterations. Panels (c) and (d) show the intensity and phase reconstruction accuracy of versus iteration time in different reconstruction processes.

The reconstruction intensity, phase correlation coefficient and RMSE of each iteration are shown in Figs. 6(a), 6(b), 6(c), and 6(d), respectively. The iterative process of the traditional FPM reconstruction algorithm is represented by the yellow curve in Fig. 6. The blue curve in Fig. 6 demonstrates the iterative reconstruction process of pcFPM. The iterative reconstruction process of tcFPM is clearly marked with the red curve, in Fig. 6. In addition, the ideal iterative reconstruction result of the known LED error is shown by the green curve in Fig. 6. In Figs. 6(a) and 6(b), the correlation coefficient of the intensity and phase of the traditional FPM iteration process (yellow line) is poor. The correlation coefficients of intensity are obviously optimized in the iterative process of pcFPM (blue line), but the result of the phase recovery is relatively poor. That the result of tcFPM (red line) is more stable, compared to traditional FPM algorithm and the pcFPM. As shown in the RMSE results of Figs. 6(c) and 6(d), it can well perceive that the result of tcFPM is more stable and accurate than the original FPM recovery algorithm and pcFPM position correction method. From what has been mentioned above, the tcFPM proposed in this paper is very close to the ideal iterative recovery process, which means that the algorithm is very stable.

### 3.2 Experiment

**Fig. 7:**
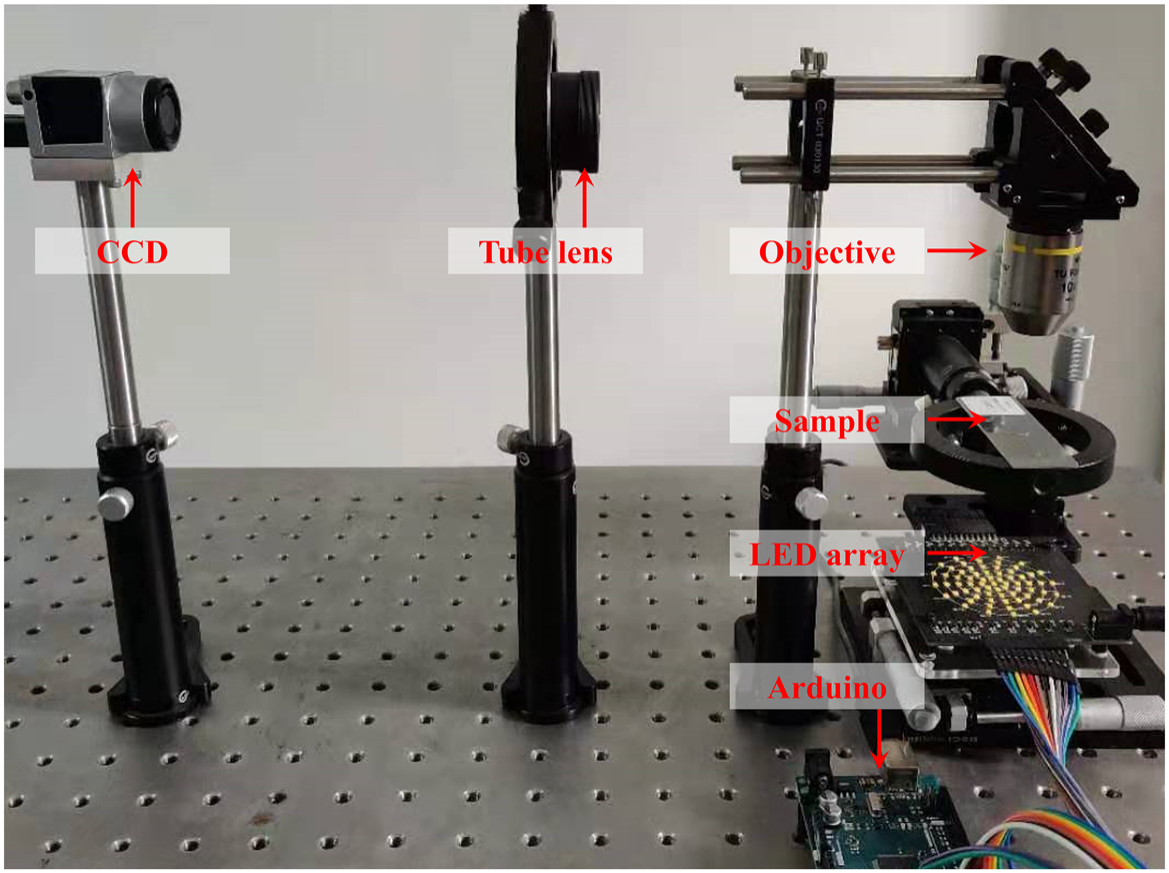
The experimental system of the tcFPM.

To prove the feasibility and performance of the tcFPM scheme, an experimental system was developed with reference to Fig. 1, as shown in Fig. 7. The main parameters of the system are as follows: the LED array (MP-1616, 1.6  mm×1.6  mm, center wavelength is 593 nm, Luminus), a microscope objective lens (10×, NA=0.3, Nikon, Japan), a tube lens (1×, focal length = 200 mm, Nikon, Japan), and a monochrome CCD camera with a maximum frame rate of 55 fps (acA2040-55  um, 2048×1536  pixels, Basler, Germany). The LED array is controlled by Arduino Mega 2560, and the CCD is triggered to collect the corresponding LR image data. Similar to the simulation, we use this system to collect 69 LR images. Without loss of generality, we choose the region of interest of the LR image to verify the performance of the system.

**Fig. 8:**
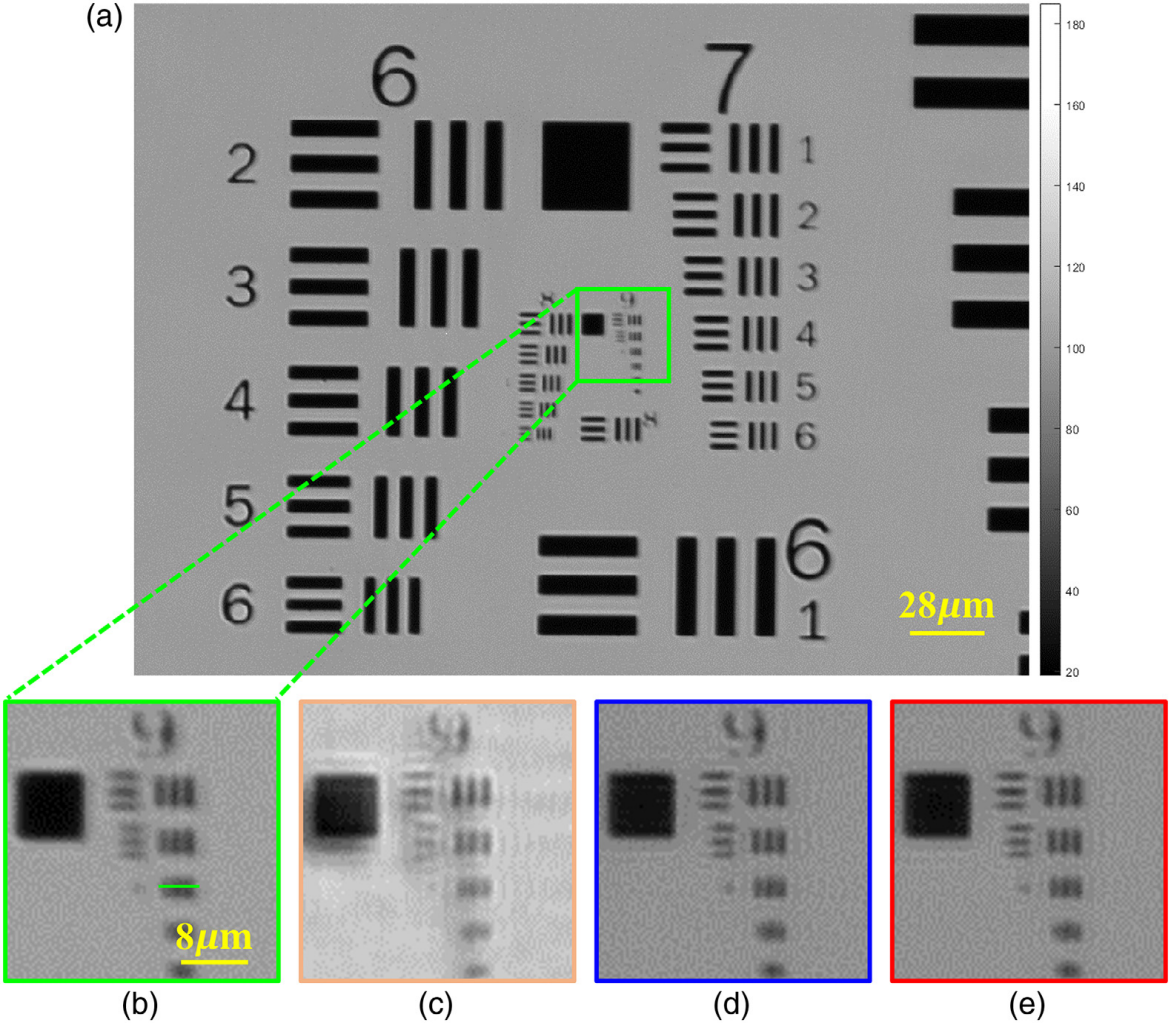
The experimental results of the USAF resolution target. (a) The full field FOV of LR image collected by CCD. The segment (b) is the enlargement of the green frame in image (a). (c) The experimental results of traditional FPM (without LED position correction). (d) The recovery results corresponding to the pcFPM scheme. (e) The corresponding reconstruction results of the tcFPM.

First, the classic USAF resolution board is selected as the experimental sample. According to the FOV of the collected LR image, a part of USAF data is selected for reconstruction operation, the results are shown in Fig. 8. The LR images are captured under the illumination of LEDs in the brightfield area, which is used as a contrast image. The interpolated result of the image is as indicated in the Fig. 8(a). Figure 8(b) shows an enlargement of the green frame area in Figure 8(a). The result of using traditional FPM recovery without correcting the LED misalignment is shown in Fig. 8(c). Figure 8(d) shows the recovery of the pcFPM program. The reconstruction of tcFPM proposed in this article is demonstrated in Fig. 8(e).

**Fig. 9:**
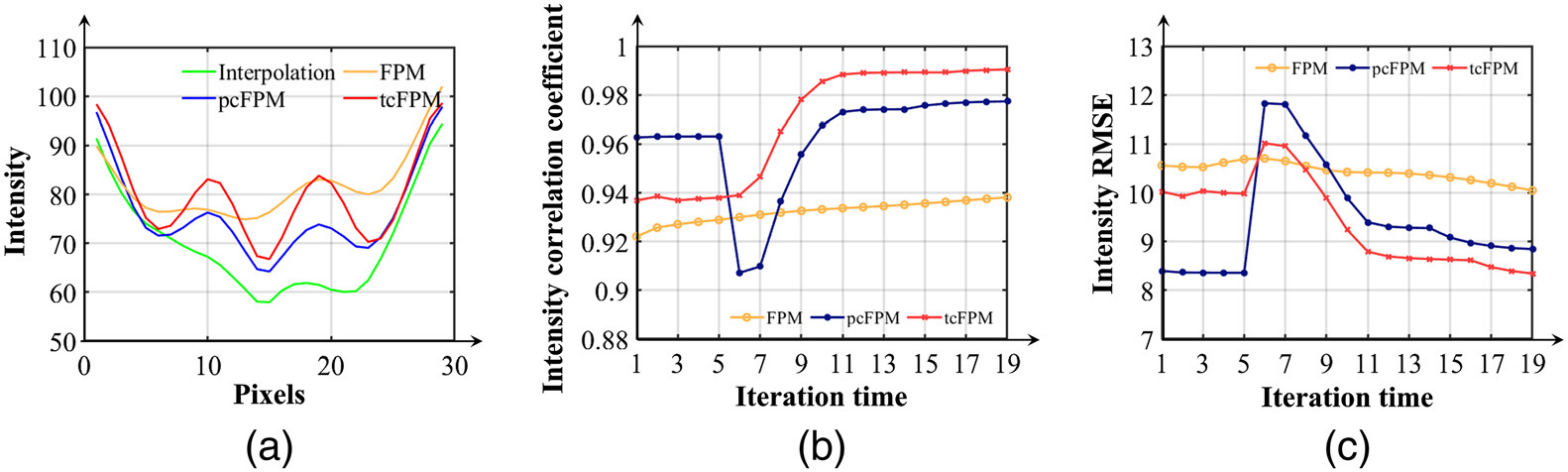
(a) The intensity curve of the third element of the ninth group [shown as the green line in Fig. 8(b)] corresponds to (b), (c), (d), and (e) in Fig. 8, respectively. (b) The intensity correlation coefficient of the reconstructed high-resolution image corresponds to three different iterative processes. (c) The intensity reconstruction accuracy of versus iteration time in different reconstruction processes.

To show the comparison of the reconstruction results, a line of intensity data of the third element of the ninth group [as demonstrated by the solid green line in Fig. 8(b)] is plotted, as shown in Fig. 9(a). The green curve in Fig. 9(a) shows that the LR intensity images collected by the CCD cannot be distinguished even after interpolation. Reconstruction through traditional FPM can improve the resolution of the result [the yellow line in the Fig. 9(a)], but the reconstructed result has low contrast and high background noise, which is the result of LED position errors. The pcFPM scheme can help improve the reconstruction resolution [the blue line in the Fig. 9(a)]. Compared with pcFPM, the tcFPM can effectively improve the resolution of the reconstructed image [the red line in the Fig. 9(a)]. The reconstruction intensity correlation coefficient and RMSE of each iteration are shown in Figs. 9(b) and 9(c), respectively. In Fig. 9(b), it can be seen that the intensity correlation coefficient of the traditional FPM iteration process [the yellow line in the Fig. 9(b)] has little improvement. Compared with FPM and pcFPM, the intensity correlation coefficient of tcFPM [the red line in the Fig. 9(b)] performs well. In addition, the iterative process [the blue line in the Fig. 9(b)] of the pcFPM scheme is unstable. The iterative process of tcFPM is relatively stable. As shown in Fig. 9(c), the RMSE of the experimental results shows that the result of tcFPM is more stable and accurate, which is compared to the FPM algorithm without LED position correction and pcFPM method. These experimental results are consistent with the previous simulation process.

**Fig. 10:**
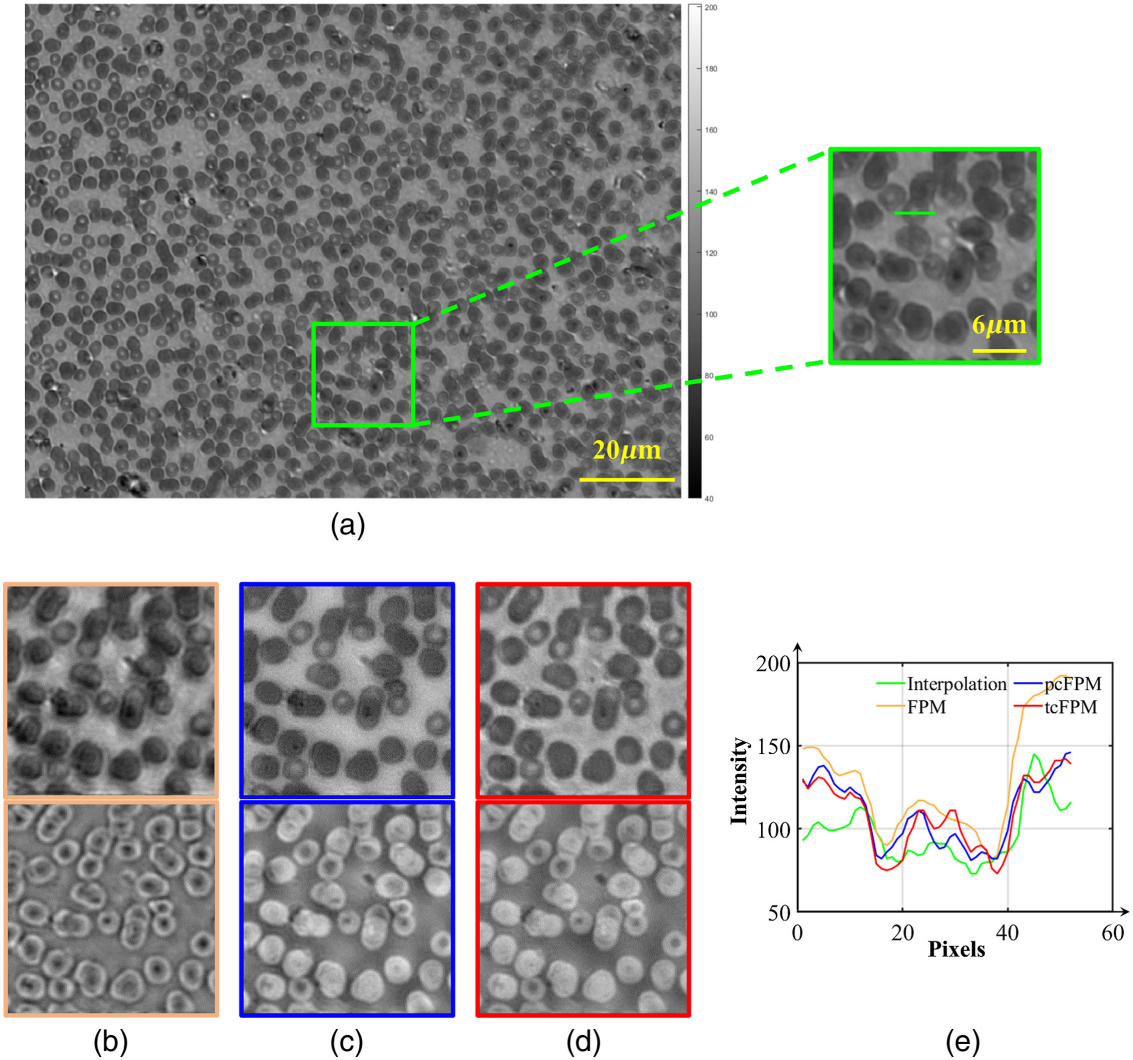
The experimental recovery image of the human blood biological sample smear. (a) LR image with full FOV. (b) The amplitude and phase of high-resolution images reconstructed by traditional FPM. (c) The AOI of the high-resolution complex amplitude image reconstructed by pcFPM. (d) The amplitude and phase of high-resolution images reconstructed by tcFPM. (e) The intensity curve of the cross-sectional profile of red blood cells.

Additionally, the stained human blood cell samples are measured and reconstructed using different algorithms. Similarly, the sample image in the imaging FOV is shown in Fig. 10(a). Figures 10(b), 10(c), and 10(d) show the AOI magnified areas of the HR intensity and phase images reconstructed by traditional FPM, pcFPM, and tcFPM, respectively. Figure 10(e) shows the intensity curve of the red blood cell cross-sectional profile at the solid green line in Figure 10(a). The result of traditional FPM restoration has serious artifacts due to the uncorrected LED position error. The results of pcFPM recovery significantly improved the impact of LED misalignment. As shown by the red intensity curve, compared with pcFPM, the cell edges in the HR image recovered by tcFPM are clearer. Overall, the reconstructed image provides more different details and eliminates obvious background noise. The results of biological samples also verified the reconstruction accuracy and stability of tcFPM.

## 4 Conclusion and Discussion

There are several comments that can be concluded from the above analysis.

a. In this proposed LED position error model, the LED position error is divided into two types. The slight offset between LEDs comes from the LED hardware design process. And, the center position of the LED array is not aligned with the optical axis. Therefore, a correction scheme for LEDs position error is proposed, which is called tcFPM.
b. To compare with existing methods, tcFPM uses the SA algorithm, which also leads to a reduction in the efficiency of system reconstruction. Therefore, how to accelerate the convergence speed of the optimization algorithm and improve the reconstruction efficiency of the proposed method is the content of further research.
c. In this proposed system, the LED position error correction process is divided into two consecutive steps. The search ranges of the two correction processes are different, which reduces the constraints of the initial position error on the optimization algorithm. If the initial position error is very large, we can also adjust the search range to solve it. However, the tcFPM would become a huge time-consuming task.

In conclusion, we have explored a tcFPM technology, which has a stable iterative process and improves the reconstruction accuracy of the complex amplitude. The correction process of LED position error is divided into two consecutive steps. First, the global offset of the LED array is preliminarily corrected. Second, the slight offset between LEDs is accurately calculated. The comparison of theoretical simulation and experimental results proves that the tcFPM can reconstruct high-quality complex amplitude results.

## References

[1] Zheng G. Horstmeyer R. Yang C., “Wide-field, high-resolution Fourier ptychographic microscopy,” Nat. Photonics 7(9), 739–745 (2013). NPAHBY 1749-4885 10.1038/nphoton.2013.187 25243016 PMC4169052

[r2] Ou X. et al., “Quantitative phase imaging via Fourier ptychographic microscopy,” Opt. Lett. 38(22), 4845 (2013). OPLEDP 0146-9592 10.1364/OL.38.004845 24322147 PMC4277232

[3] Pan A. Zuo C. Yao B., “High-resolution and large field-of-view Fourier ptychographic microscopy and its applications in biomedicine,” Rep. Prog. Phys. 83(9), 096101 (2020). RPPHAG 0034-4885 10.1088/1361-6633/aba6f0 32679569

[4] Ou X. et al., “High numerical aperture Fourier ptychography: principle, implementation and characterization,” Opt. Express 23(3), 3472 (2015). OPEXFF 1094-4087 10.1364/OE.23.003472 25836203 PMC5802253

[5] Tian L. et al., “Multiplexed coded illumination for Fourier ptychography with an LED array microscope,” Biomed. Opt. Express 5(7), 2376 (2014). BOEICL 2156-7085 10.1364/BOE.5.002376 25071971 PMC4102371

[6] Sun J. et al., “Sampling criteria for Fourier ptychographic microscopy in object space and frequency space,” Opt. Express 24(14), 15765 (2016). OPEXFF 1094-4087 10.1364/OE.24.015765 27410848

[7] Zheng G. et al., “Characterization of spatially varying aberrations for wide field-of-view microscopy,” Opt. Express 21(13), 15131–15143 (2013). 10.1364/OE.21.015131 23842300 PMC3724395

[8] Ou X. Zheng G. Yang C., “Embedded pupil function recovery for Fourier ptychographic microscopy,” Opt. Express 22, 4960–4972 (2014). 10.1364/OE.22.004960 24663835 PMC4086333

[9] Zuo C. Sun J. Chen Q., “Adaptive step-size strategy for noise-robust Fourier ptychographic microscopy,” Opt. Express 24(18), 20724 (2016). OPEXFF 1094-4087 10.1364/OE.24.020724 27607676

[r10] Zhang Y. Jiang W. Dai Q., “Nonlinear optimization approach for Fourier ptychographic microscopy,” Opt. Express 23(26), 33822 (2015). OPEXFF 1094-4087 10.1364/OE.23.033822 26832043

[r11] Zhang Y. Pan A. Lei M., “Data preprocessing methods for robust Fourier ptychographic microscopy,” Opt. Eng 56(12), 123107 (2017). 10.1117/1.OE.56.12.123107

[12] Fan Y. et al., “Adaptive denoising method for Fourier ptychographic microscopy,” Optics Communications 404, 23–31 (2017). OPCOB8 0030-4018 10.1016/j.optcom.2017.05.026

[13] Pan A. et al., “Vignetting effect in Fourier ptychographic microscopy,” Opt. Lasers Eng. 120, 40–48 (2019). 10.1016/j.optlaseng.2019.02.015

[14] Dong S. et al., “Sparsely sampled Fourier ptychography,” Opt. Express 22(5), 5455 (2014). OPEXFF 1094-4087 10.1364/OE.22.005455 24663886

[15] Bian Z. Dong S. Zheng G., “Adaptive system correction for robust Fourier ptychographic imaging,” Opt. Express 21(26), 32400 (2013). OPEXFF 1094-4087 10.1364/OE.21.032400 24514833

[16] Pan A. et al., “Subwavelength resolution Fourier ptychography with hemispherical digital condensers,” Opt. Express 26(18), 23119 (2018). OPEXFF 1094-4087 10.1364/OE.26.023119 30184967

[17] Yeh L.-H. et al., “Experimental robustness of Fourier ptychography phase retrieval algorithms,” Opt. Express 23(26), 33214 (2015). OPEXFF 1094-4087 10.1364/OE.23.033214 26831989

[r18] Liu J. et al., “Stable and robust frequency domain position compensation strategy for Fourier ptychographic microscopy,” Opt. Express 25(23), 28053 (2017). OPEXFF 1094-4087 10.1364/OE.25.028053

[r19] Chen S. et al., “Random positional deviations correction for each LED via ePIE in Fourier ptychographic microscopy,” IEEE Access 6, 33399–33409 (2018). 10.1109/ACCESS.2018.2849010

[r20] Zhang J. et al., “Precise brightfield localization alignment for Fourier ptychographic microscopy,” IEEE Photonics J. 10(1), 14 (2018). 10.1109/JPHOT.2017.2780189

[r21] Eckert R. Phillips Z. F. Waller L., “Efficient illumination angle self-calibration in Fourier ptychography,” Appl. Opt. 57(19), 5434 (2018). APOPAI 0003-6935 10.1364/AO.57.005434 30117837

[r22] Zhou A. et al., “Fast and robust misalignment correction of Fourier ptychographic microscopy for full field of view reconstruction,” Opt. Express 26(18), 23661 (2018). OPEXFF 1094-4087 10.1364/OE.26.023661 30184864

[r23] Sun J. et al., “Efficient positional misalignment correction method for Fourier ptychographic microscopy,” Biomed. Opt. Express 7(4), 1336 (2016). BOEICL 2156-7085 10.1364/BOE.7.001336 27446659 PMC4929645

[r24] Pan A. et al., “System calibration method for Fourier ptychographic microscopy,” J. Biomed. Opt 22(09), 096005 (2017). JBOPFO 1083-3668 10.1117/1.JBO.22.9.096005 28901054

[25] Zhang J. et al., “A positional misalignment correction method for Fourier ptychographic microscopy based on the quasi-Newton method with a global optimization module,” Opt. Commun. 452, 296–305 (2019). OPCOB8 0030-4018 10.1016/j.optcom.2019.07.046

[26] Guo K. et al., “Optimization of sampling pattern and the design of Fourier ptychographic illuminator,” Opt. Express 23(5), 6171 (2015). OPEXFF 1094-4087 10.1364/OE.23.006171 25836839

[27] Tao X. et al., “Tunable-illumination for laser Fourier ptychographic microscopy based on a background noise-reducing system,” Opt. Commun. 468, 125764 (2020). OPCOB8 0030-4018 10.1016/j.optcom.2020.125764

